# Tris{aqua­bis[3-(2-pyrid­yl)-1*H*-pyrazole]copper(II)} di-μ_9_-arsenato-hexa­triaconta-μ_2_-oxido-octa­deca­oxidoocta­deca­molybdate(VI)

**DOI:** 10.1107/S160053681000156X

**Published:** 2010-01-16

**Authors:** Xiutang Zhang, Peihai Wei, Congwen Shi, Bin Li, Bo Hu

**Affiliations:** a Advanced Material Institute of Research, Department of Chemistry and Chemical Engineering, ShanDong Institute of Education, Jinan 250013, People’s Republic of China; b College of Chemistry and Chemical Engineering, Liaocheng University, Liaocheng 252059, People’s Republic of China

## Abstract

The title compound, [Cu(C_8_H_7_N_3_)_2_(H_2_O)]_3_[As_2_Mo_18_O_62_], consists of two subunits, *viz*. an α-Dawson-type [As_2_Mo_18_O_62_]^6−^ anion and a complex [Cu(C_8_H_7_N_3_)_2_(H_2_O)]^2+^ cation. The copper(II) ion (site symmetry .2) is penta­coordinated in a distorted square-pyramidal manner by four N atoms from two chelating 3-(2-pyrid­yl)pyrazole ligands in equatorial positions and one water mol­ecule in the apical position. In the heteropolyanion, two O atoms of the AsO_4_ group (3. symmetry) are equally disordered about the threefold rotation axis. N—H⋯O and O—H⋯O hydrogen bonding between the neutral mol­ecules and the water mol­ecules leads to a consolidation of the structure.

## Related literature

For background to polyoxometalates, see: Pope & Müller (1991 ▶). For polyoxometalates modified with amines, see: Zhang, Dou *et al.* (2009 ▶); Zhang, Wei, Shi *et al.* (2010 ▶); Zhang, Wei *et al.* (2009 ▶); Zhang, Yuan *et al.* (2010 ▶). Zhang, Wei, Zhu *et al.* (2010 ▶). For another α-Dawson-type anion, see: Li *et al.* (2007 ▶).
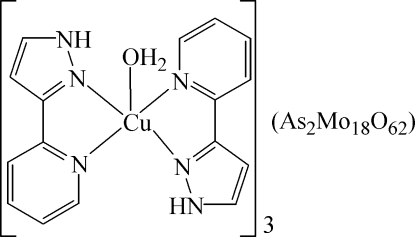


## Experimental

### 

#### Crystal data

[Cu(C_8_H_7_N_3_)_2_(H_2_O)]_3_[As_2_Mo_18_O_62_]
*M* *_r_* = 3984.45
Hexagonal, 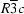

*a* = 21.967 (3) Å
*c* = 34.411 (7) Å
*V* = 14380 (4) Å^3^
*Z* = 6
Mo *K*α radiation
μ = 3.72 mm^−1^
*T* = 293 K
0.12 × 0.10 × 0.08 mm

#### Data collection

Bruker APEXII CCD diffractometer
Absorption correction: multi-scan (*SADABS*; Bruker, 2001 ▶) *T*_min_ = 0.664, *T*_max_ = 0.755
25458 measured reflections
2750 independent reflections
2053 reflections with *I* > 2σ(*I*)
*R*_int_ = 0.085

#### Refinement

*R*[*F* ^2^ > 2σ(*F* ^2^)] = 0.046
*wR*(*F* ^2^) = 0.123
*S* = 1.00
2750 reflections
254 parameters
14 restraints
H atoms treated by a mixture of independent and constrained refinement
Δρ_max_ = 2.25 e Å^−3^
Δρ_min_ = −1.15 e Å^−3^

### 

Data collection: *APEX2* (Bruker, 2004 ▶); cell refinement: *SAINT-Plus* (Bruker, 2001 ▶); data reduction: *SAINT-Plus*; program(s) used to solve structure: *SHELXS97* (Sheldrick, 2008 ▶); program(s) used to refine structure: *SHELXL97* (Sheldrick, 2008 ▶); molecular graphics: *SHELXTL* (Sheldrick, 2008 ▶); software used to prepare material for publication: *SHELXTL*.

## Supplementary Material

Crystal structure: contains datablocks global, I. DOI: 10.1107/S160053681000156X/wm2290sup1.cif

Structure factors: contains datablocks I. DOI: 10.1107/S160053681000156X/wm2290Isup2.hkl

Additional supplementary materials:  crystallographic information; 3D view; checkCIF report

## Figures and Tables

**Table 1:** Selected bond lengths (Å)

As1—O10*A*	1.653 (10)
As1—O10*B*	1.677 (10)
As1—O1	1.728 (9)
Cu1—N3	1.984 (7)
Cu1—N1	1.985 (8)
Cu1—O11	2.29 (2)

**Table 2:** Hydrogen-bond geometry (Å, °)

*D*—H⋯*A*	*D*—H	H⋯*A*	*D*⋯*A*	*D*—H⋯*A*
N2—H2*A*⋯O6^i^	0.86	2.27	3.097 (13)	162
O11—H1*W*⋯O3^ii^	0.84 (8)	2.69 (11)	2.860 (10)	94 (8)

Symmetry codes: (i) 
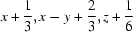
; (ii) 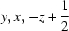.

**Fig. 1.**
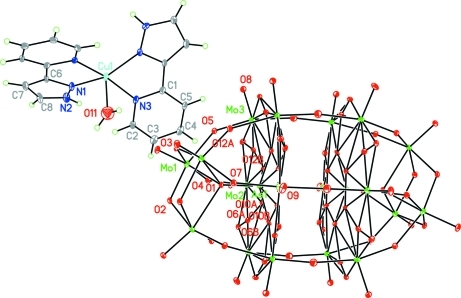
The cation and anion of the title compound with the atom-labelling scheme. Displacement ellipsoids are drawn at the 30% probability level; H atoms are given as spheres of arbitrary radius.

**Fig. 2.**
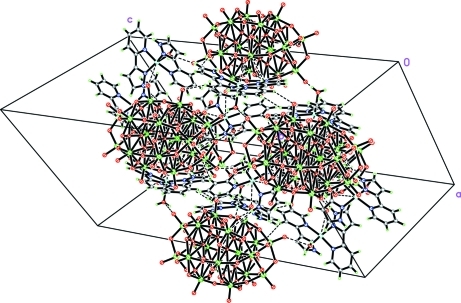
The crystal packing of the title compound, displayed with hydrogen bonds as dashed lines.

## supplementary crystallographic information

### Comment

The design and synthesis of polyoxometalates has attracted continuous research interest not only because of their appealing structural and topological novelties, but also due to their interesting optical, electronic, magnetic, and catalytic properties, as well as their potential medical applications (Pope *et al.*, 1991). In our group, organic amines, such as 3-(2-pyridyl)pyrazole and pyrazine, are used to effectively modify polyoxomolybdates under hydrothermal condictions (Zhang, Dou *et al.*, 2009; Zhang, Wei *et al.*, 2009). Here, we describe the synthesis and structural characterization of the title compound.

As shown in Figure 1, the title compound consists of two subunits, *viz.* of an α-Dawson-type [As_2_Mo_18_O_62_]^6-^ anion (Li *et al.*, 2007) and a complex [Cu(H_2_O)(C_8_H_7_N_3_)_2_]^2+^ cation. The copper ion is penta-coordinated in a distorted square-pyramidal geometry by four N atoms from two 3-(2-pyridyl)pyrazole ligands and by one water molecule. The Cu—N bond lengths are in the range of 1.984 (7)—1.985 (8) Å and the Cu—O bond length is 2.29 (2) Å. In the heteropolyanion, there are four kinds of oxygen atoms according to their coordination manner: (i) 18 terminal O atoms bonded to one Mo atom with their Mo—O distances in the range of 1.651 (6)—1.690 (6) Å; (ii) 36 µ_2_ O atoms, the Mo—O distances in the range of 1.797 (5)—2.117 (5) Å; (iii) six µ_3_ O atoms shared by one As and two Mo atoms, the Mo—O distances varying from 1.653 (8) to 2.359 (1) Å; (iv) two µ_4_ O atoms which are coordinated to one As atom and three Mo atoms, Mo—O distances are between 1.728 (7) and 2.341 (7) Å, respectively. The resulting MoO_6_ octahedra are considerably distorted. The AsO_4_ group is disordered about a threefold rotation axis and exhibuts two sets of short As—O bond lenghts to the disordered O atoms (50% occupation) and one longer As—O bond. N—H···O and O—H···O hydrogen bonding between the neutral molecules and the water molecules leads to a consolidation of the structure (Fig. 2; Table 2) which also contains accessible voids of ca. 136 Å^3^.

### Experimental

A mixture of 3-(2-pyridyl)pyrazole (1 mmoL 0.14 g), sodium molybdate (2 mmoL, 0.48 g), sodium arsenate (0.2 mmoL, 0.08 g) and copper dichloride dihydrate (1 mmoL, 0.28 g) in 14 ml distilled water was sealed in a 25 ml Teflon-lined stainless steel autoclave and was kept at 433 K for three days. Blue crystals suitable for the X-ray experiment were obtained. Anal. Calc. for C_48_H_48_As_2_Cu_3_Mo_18_N_18_O_65_: C 14.46, H 1.20, N 6.32%; Found: C 14.24, H 1.01, N 6.23%.

### Refinement

All hydrogen atoms bound to carbon were refined using a riding model with distance C—H = 0.93 Å, *U*_iso_ = 1.2*U*_eq_ (C) for aromatic atoms. The H atoms of the water molecule were located from difference density maps and were refined with d(O—H) = 0.83 (2) Å, and with a fixed *U*_iso_ of 0.80 Å^2^. In the AsO_4_ unit, two oxygen atoms (O6 and O10) are disordered around a threefold rotation axis. Both positions were refined with split positions and an occupancy ratio of 1:1. In the final difference Fourier map the highest peak is 3.20 Å from atom O2 and the deepest hole is 0.67 A Å from atom O11. The highest peak is located in the voids of the crystal structure and may be associated with an additional water molecule. However, refinement of this position did not result in a reasonable model. Hence this position was excluded from the final refinement.

### Crystal data

**Table:**

[Cu(C_8_H_7_N_3_)_2_(H_2_O)]_3_[As_2_Mo_18_O_62_]	*D*_x_ = 2.761 Mg m^−^^3^
*M**_r_* = 3984.45	Mo *K*α radiation, λ = 0.71073 Å
Hexagonal, *R*3*c*	Cell parameters from 2750 reflections
Hall symbol: -R 3 2"c	θ = 1.6–25.0°
*a* = 21.967 (3) Å	µ = 3.72 mm^−^^1^
*c* = 34.411 (7) Å	*T* = 293 K
*V* = 14380 (4) Å^3^	Block, blue
*Z* = 6	0.12 × 0.10 × 0.08 mm
*F*(000) = 11346	

### Data collection

**Table:**

Bruker APEXII CCD diffractometer	2750 independent reflections
Radiation source: fine-focus sealed tube	2053 reflections with *I* > 2σ(*I*)
graphite	*R*_int_ = 0.085
phi and ω scans	θ_max_ = 25.0°, θ_min_ = 1.6°
Absorption correction: multi-scan (*SADABS*; Bruker, 2001)	*h* = −25→26
*T*_min_ = 0.664, *T*_max_ = 0.755	*k* = −26→26
25458 measured reflections	*l* = −40→39

### Refinement

**Table:**

Refinement on *F*^2^	Primary atom site location: structure-invariant direct methods
Least-squares matrix: full	Secondary atom site location: difference Fourier map
*R*[*F*^2^ > 2σ(*F*^2^)] = 0.046	Hydrogen site location: inferred from neighbouring sites
*wR*(*F*^2^) = 0.123	H atoms treated by a mixture of independent and constrained refinement
*S* = 1.00	*w* = 1/[σ^2^(*F*_o_^2^) + (0.063*P*)^2^ + 278.6957*P*] where *P* = (*F*_o_^2^ + 2*F*_c_^2^)/3
2750 reflections	(Δ/σ)_max_ = 0.001
254 parameters	Δρ_max_ = 2.25 e Å^−^^3^
14 restraints	Δρ_min_ = −1.15 e Å^−^^3^

### Special details

**Table:**

Geometry. All e.s.d.'s (except the e.s.d. in the dihedral angle between two l.s. planes) are estimated using the full covariance matrix. The cell e.s.d.'s are taken into account individually in the estimation of e.s.d.'s in distances, angles and torsion angles; correlations between e.s.d.'s in cell parameters are only used when they are defined by crystal symmetry. An approximate (isotropic) treatment of cell e.s.d.'s is used for estimating e.s.d.'s involving l.s. planes.
Refinement. Refinement of *F*^2^ against ALL reflections. The weighted *R*-factor *wR* and goodness of fit *S* are based on *F*^2^, conventional *R*-factors *R* are based on *F*, with *F* set to zero for negative *F*^2^. The threshold expression of *F*^2^ > σ(*F*^2^) is used only for calculating *R*-factors(gt) *etc*. and is not relevant to the choice of reflections for refinement. *R*-factors based on *F*^2^ are statistically about twice as large as those based on *F*, and *R*- factors based on ALL data will be even larger.

### Fractional atomic coordinates and isotropic or equivalent isotropic displacement parameters (Å^2^)

**Table:**

	*x*	*y*	*z*	*U*_iso_*/*U*_eq_	Occ. (<1)
As1	0.0000	0.0000	0.05884 (4)	0.0161 (3)	
C1	0.2641 (5)	0.2974 (5)	0.1696 (3)	0.039 (2)	
C2	0.1872 (5)	0.3163 (5)	0.2056 (3)	0.042 (2)	
H2	0.1698	0.3199	0.2296	0.050*	
C3	0.1552 (7)	0.3216 (5)	0.1730 (3)	0.055 (3)	
H3	0.1192	0.3321	0.1747	0.066*	
C4	0.1768 (7)	0.3112 (5)	0.1380 (3)	0.055 (3)	
H4	0.1534	0.3113	0.1155	0.065*	
C5	0.2326 (6)	0.3008 (5)	0.1359 (3)	0.042 (3)	
H5	0.2493	0.2960	0.1119	0.050*	
C6	0.2831 (5)	0.3216 (5)	0.3283 (2)	0.037 (2)	
C7	0.2761 (7)	0.3645 (7)	0.3554 (4)	0.066 (3)	
H7	0.2809	0.3636	0.3822	0.079*	
C8	0.2605 (7)	0.4092 (6)	0.3343 (3)	0.067 (4)	
H8	0.2523	0.4438	0.3442	0.080*	
Cu1	0.28491 (9)	0.28491 (9)	0.2500	0.0444 (5)	
Mo1	0.06070 (4)	0.10373 (4)	0.14523 (2)	0.0293 (2)	
Mo2	0.03113 (4)	0.17477 (4)	0.05152 (2)	0.0270 (2)	
Mo3	0.17292 (4)	0.14366 (4)	0.05871 (2)	0.0298 (2)	
N1	0.2731 (5)	0.3393 (5)	0.2924 (2)	0.045 (2)	
N2	0.2599 (5)	0.3921 (5)	0.2969 (3)	0.057 (3)	
H2A	0.2518	0.4126	0.2779	0.068*	
N3	0.2426 (4)	0.3063 (4)	0.2048 (2)	0.0381 (19)	
O1	0.0000	0.0000	0.1090 (3)	0.023 (2)	
O2	−0.0297 (3)	0.0513 (3)	0.16857 (15)	0.0250 (12)	
O3	0.1016 (3)	0.1636 (3)	0.18082 (17)	0.0336 (15)	
O4	0.0331 (3)	0.1452 (3)	0.10985 (16)	0.0261 (13)	
O5	0.1395 (3)	0.1213 (3)	0.11134 (16)	0.0271 (13)	
O6	−0.0665 (5)	0.1212 (5)	0.0577 (3)	0.075 (2)	
O7	0.0438 (3)	0.2531 (3)	0.06422 (17)	0.0344 (15)	
O8	0.2534 (3)	0.2085 (3)	0.06733 (18)	0.0412 (17)	
O9	0.0315 (4)	0.1816 (3)	−0.00079 (17)	0.0445 (18)	
O10A	−0.0288 (5)	0.0530 (5)	0.0445 (3)	0.019 (2)	0.50
O10B	−0.0832 (5)	−0.0297 (5)	0.0446 (3)	0.016 (2)	0.50
O11	0.1808 (10)	0.1808 (10)	0.2500	0.142 (2)	
O12A	0.1344 (7)	0.2076 (7)	0.0591 (4)	0.030 (3)	0.50
O12B	0.1111 (7)	0.1708 (8)	0.0481 (4)	0.027 (3)	0.50
H1W	0.1487 (19)	0.179 (2)	0.264 (3)	0.080*	

### Atomic displacement parameters (Å^2^)

**Table:**

	*U*^11^	*U*^22^	*U*^33^	*U*^12^	*U*^13^	*U*^23^
As1	0.0173 (4)	0.0173 (4)	0.0136 (7)	0.0086 (2)	0.000	0.000
C1	0.043 (6)	0.025 (5)	0.034 (6)	0.005 (4)	−0.004 (4)	0.000 (4)
C2	0.058 (7)	0.042 (6)	0.032 (6)	0.030 (5)	−0.006 (5)	−0.003 (4)
C3	0.081 (9)	0.041 (6)	0.051 (7)	0.038 (6)	−0.015 (6)	−0.005 (5)
C4	0.082 (9)	0.030 (6)	0.055 (8)	0.031 (6)	−0.018 (6)	−0.004 (5)
C5	0.063 (7)	0.032 (5)	0.020 (5)	0.016 (5)	−0.011 (4)	−0.002 (4)
C6	0.045 (6)	0.044 (6)	0.018 (5)	0.019 (5)	0.004 (4)	0.000 (4)
C7	0.076 (9)	0.065 (8)	0.042 (7)	0.025 (7)	0.010 (6)	0.006 (6)
C8	0.099 (10)	0.058 (8)	0.040 (7)	0.036 (7)	0.021 (7)	−0.002 (6)
Cu1	0.0590 (9)	0.0590 (9)	0.0215 (9)	0.0344 (10)	−0.0019 (4)	0.0019 (4)
Mo1	0.0391 (5)	0.0246 (4)	0.0214 (4)	0.0139 (4)	−0.0087 (3)	−0.0058 (3)
Mo2	0.0362 (5)	0.0200 (4)	0.0270 (4)	0.0156 (3)	0.0013 (3)	0.0008 (3)
Mo3	0.0201 (4)	0.0244 (4)	0.0388 (5)	0.0067 (3)	−0.0063 (3)	−0.0045 (3)
N1	0.065 (6)	0.048 (5)	0.022 (4)	0.028 (5)	0.000 (4)	−0.001 (4)
N2	0.086 (7)	0.041 (5)	0.044 (6)	0.033 (5)	0.007 (5)	0.016 (4)
N3	0.048 (5)	0.028 (4)	0.025 (4)	0.009 (4)	−0.007 (4)	0.003 (3)
O1	0.027 (3)	0.027 (3)	0.016 (5)	0.0133 (16)	0.000	0.000
O2	0.028 (3)	0.029 (3)	0.016 (3)	0.013 (3)	0.003 (2)	−0.001 (2)
O3	0.044 (4)	0.028 (3)	0.023 (3)	0.013 (3)	−0.008 (3)	−0.006 (3)
O4	0.028 (3)	0.027 (3)	0.025 (3)	0.015 (3)	0.006 (2)	0.005 (2)
O5	0.030 (3)	0.030 (3)	0.024 (3)	0.017 (3)	0.001 (2)	0.001 (2)
O6	0.074 (3)	0.075 (3)	0.073 (3)	0.0349 (15)	−0.0029 (10)	0.0033 (10)
O7	0.048 (4)	0.025 (3)	0.030 (3)	0.018 (3)	0.001 (3)	−0.001 (3)
O8	0.024 (3)	0.048 (4)	0.035 (4)	0.005 (3)	−0.002 (3)	−0.014 (3)
O9	0.087 (5)	0.030 (4)	0.022 (3)	0.034 (4)	0.001 (3)	0.002 (3)
O10A	0.015 (5)	0.022 (6)	0.020 (6)	0.009 (5)	0.003 (4)	0.001 (4)
O10B	0.017 (5)	0.012 (5)	0.020 (6)	0.009 (4)	0.009 (4)	0.001 (4)
O11	0.142 (2)	0.142 (2)	0.142 (2)	0.0707 (13)	−0.0005 (7)	0.0005 (7)
O12A	0.029 (8)	0.023 (8)	0.037 (9)	0.012 (7)	0.001 (6)	0.001 (6)
O12B	0.028 (8)	0.029 (8)	0.022 (7)	0.012 (7)	0.004 (6)	−0.005 (6)

### Geometric parameters (Å, °)

**Table:**

As1—O10A^i^	1.653 (10)	Mo1—O1	2.341 (5)
As1—O10A	1.653 (10)	Mo2—O7	1.658 (6)
As1—O10A^ii^	1.653 (10)	Mo2—O12B	1.806 (13)
As1—O10B	1.677 (10)	Mo2—O9	1.806 (6)
As1—O10B^i^	1.677 (10)	Mo2—O6	1.872 (9)
As1—O10B^ii^	1.677 (10)	Mo2—O12A	2.024 (13)
As1—O1	1.728 (9)	Mo2—O4	2.117 (5)
C1—N3	1.350 (12)	Mo2—O10B^ii^	2.309 (9)
C1—C5	1.371 (13)	Mo2—O10A	2.330 (10)
C1—C6^iii^	1.447 (14)	Mo3—O8	1.651 (6)
C2—N3	1.342 (12)	Mo3—O12B	1.772 (13)
C2—C3	1.359 (14)	Mo3—O6^ii^	1.878 (9)
C2—H2	0.9300	Mo3—O5	1.923 (6)
C3—C4	1.355 (16)	Mo3—O12A	1.970 (13)
C3—H3	0.9300	Mo3—O9^iv^	2.000 (6)
C4—C5	1.358 (15)	Mo3—O10B^ii^	2.325 (9)
C4—H4	0.9300	Mo3—O10A^ii^	2.359 (10)
C5—H5	0.9300	N1—N2	1.337 (12)
C6—N1	1.345 (11)	N2—H2A	0.8600
C6—C7	1.389 (15)	O1—Mo1^i^	2.341 (5)
C6—C1^iii^	1.447 (14)	O1—Mo1^ii^	2.341 (5)
C7—C8	1.395 (17)	O2—Mo1^i^	2.053 (5)
C7—H7	0.9300	O6—Mo3^i^	1.878 (9)
C8—N2	1.339 (13)	O9—Mo3^v^	2.000 (6)
C8—H8	0.9300	O10A—O10B^ii^	1.582 (13)
Cu1—N3^iii^	1.984 (7)	O10A—O10B	1.599 (13)
Cu1—N3	1.984 (7)	O10A—Mo3^i^	2.359 (10)
Cu1—N1	1.985 (8)	O10B—O10A^i^	1.582 (13)
Cu1—N1^iii^	1.985 (8)	O10B—O12B^i^	1.698 (17)
Cu1—O11	2.29 (2)	O10B—Mo2^i^	2.309 (9)
Mo1—O3	1.690 (6)	O10B—Mo3^i^	2.325 (9)
Mo1—O4	1.797 (5)	O11—H1W	0.84 (8)
Mo1—O2	1.904 (5)	O12A—O12B	0.804 (14)
Mo1—O5	1.959 (6)	O12B—O10B^ii^	1.698 (17)
Mo1—O2^ii^	2.053 (5)		
			
O10A^i^—As1—O10A	111.5 (3)	O7—Mo2—O10A	157.6 (3)
O10A^i^—As1—O10A^ii^	111.5 (3)	O12B—Mo2—O10A	86.7 (5)
O10A—As1—O10A^ii^	111.5 (3)	O9—Mo2—O10A	88.2 (3)
O10A^i^—As1—O10B	56.7 (4)	O6—Mo2—O10A	58.5 (4)
O10A—As1—O10B	57.4 (4)	O12A—Mo2—O10A	108.2 (5)
O10A^ii^—As1—O10B	145.7 (5)	O4—Mo2—O10A	80.3 (3)
O10A^i^—As1—O10B^i^	57.4 (4)	O10B^ii^—Mo2—O10A	39.9 (3)
O10A—As1—O10B^i^	145.7 (5)	O8—Mo3—O12B	114.7 (5)
O10A^ii^—As1—O10B^i^	56.7 (4)	O8—Mo3—O6^ii^	100.9 (4)
O10B—As1—O10B^i^	111.8 (3)	O12B—Mo3—O6^ii^	143.7 (6)
O10A^i^—As1—O10B^ii^	145.7 (5)	O8—Mo3—O5	99.1 (3)
O10A—As1—O10B^ii^	56.7 (4)	O12B—Mo3—O5	91.2 (4)
O10A^ii^—As1—O10B^ii^	57.4 (4)	O6^ii^—Mo3—O5	90.1 (3)
O10B—As1—O10B^ii^	111.8 (3)	O8—Mo3—O12A	92.3 (5)
O10B^i^—As1—O10B^ii^	111.8 (3)	O12B—Mo3—O12A	24.1 (4)
O10A^i^—As1—O1	107.4 (4)	O6^ii^—Mo3—O12A	166.7 (5)
O10A—As1—O1	107.4 (4)	O5—Mo3—O12A	86.0 (4)
O10A^ii^—As1—O1	107.4 (4)	O8—Mo3—O9^iv^	95.5 (3)
O10B—As1—O1	107.0 (3)	O12B—Mo3—O9^iv^	80.4 (5)
O10B^i^—As1—O1	107.0 (3)	O6^ii^—Mo3—O9^iv^	89.6 (4)
O10B^ii^—As1—O1	107.0 (3)	O5—Mo3—O9^iv^	165.2 (2)
N3—C1—C5	122.0 (10)	O12A—Mo3—O9^iv^	90.9 (5)
N3—C1—C6^iii^	113.1 (8)	O8—Mo3—O10B^ii^	161.3 (3)
C5—C1—C6^iii^	124.9 (9)	O12B—Mo3—O10B^ii^	46.6 (5)
N3—C2—C3	123.3 (10)	O6^ii^—Mo3—O10B^ii^	97.8 (4)
N3—C2—H2	118.4	O5—Mo3—O10B^ii^	82.7 (3)
C3—C2—H2	118.4	O12A—Mo3—O10B^ii^	69.1 (5)
C4—C3—C2	118.6 (11)	O9^iv^—Mo3—O10B^ii^	82.7 (3)
C4—C3—H3	120.7	O8—Mo3—O10A^ii^	158.5 (3)
C2—C3—H3	120.7	O12B—Mo3—O10A^ii^	86.2 (5)
C3—C4—C5	119.9 (11)	O6^ii^—Mo3—O10A^ii^	57.9 (4)
C3—C4—H4	120.1	O5—Mo3—O10A^ii^	84.9 (3)
C5—C4—H4	120.1	O12A—Mo3—O10A^ii^	109.0 (5)
C4—C5—C1	119.1 (10)	O9^iv^—Mo3—O10A^ii^	82.5 (3)
C4—C5—H5	120.4	O10B^ii^—Mo3—O10A^ii^	39.9 (3)
C1—C5—H5	120.4	N2—N1—C6	106.6 (8)
N1—C6—C7	109.3 (10)	N2—N1—Cu1	139.1 (7)
N1—C6—C1^iii^	115.9 (8)	C6—N1—Cu1	114.2 (7)
C7—C6—C1^iii^	134.8 (9)	N1—N2—C8	112.1 (9)
C6—C7—C8	106.0 (10)	N1—N2—H2A	124.0
C6—C7—H7	127.0	C8—N2—H2A	124.0
C8—C7—H7	127.0	C2—N3—C1	116.9 (8)
N2—C8—C7	106.1 (11)	C2—N3—Cu1	126.5 (7)
N2—C8—H8	127.0	C1—N3—Cu1	115.5 (7)
C7—C8—H8	127.0	As1—O1—Mo1^i^	122.13 (18)
N3^iii^—Cu1—N3	166.7 (4)	As1—O1—Mo1	122.13 (18)
N3^iii^—Cu1—N1	80.7 (3)	Mo1^i^—O1—Mo1	94.3 (2)
N3—Cu1—N1	102.5 (3)	As1—O1—Mo1^ii^	122.13 (18)
N3^iii^—Cu1—N1^iii^	102.5 (3)	Mo1^i^—O1—Mo1^ii^	94.3 (2)
N3—Cu1—N1^iii^	80.7 (3)	Mo1—O1—Mo1^ii^	94.3 (2)
N1—Cu1—N1^iii^	152.7 (5)	Mo1—O2—Mo1^i^	120.4 (3)
N3^iii^—Cu1—O11	83.4 (2)	Mo1—O4—Mo2	149.5 (3)
N3—Cu1—O11	83.4 (2)	Mo3—O5—Mo1	142.9 (3)
N1—Cu1—O11	103.6 (3)	Mo2—O6—Mo3^i^	144.3 (5)
N1^iii^—Cu1—O11	103.6 (3)	Mo2—O9—Mo3^v^	170.5 (4)
O3—Mo1—O4	106.2 (3)	O10B^ii^—O10A—O10B	121.6 (9)
O3—Mo1—O2	98.8 (3)	O10B^ii^—O10A—As1	62.4 (5)
O4—Mo1—O2	94.5 (2)	O10B—O10A—As1	62.0 (5)
O3—Mo1—O5	101.8 (3)	O10B^ii^—O10A—Mo2	69.3 (5)
O4—Mo1—O5	89.2 (2)	O10B—O10A—Mo2	167.6 (7)
O2—Mo1—O5	157.1 (2)	As1—O10A—Mo2	125.6 (5)
O3—Mo1—O2^ii^	95.3 (2)	O10B^ii^—O10A—Mo3^i^	164.3 (7)
O4—Mo1—O2^ii^	158.1 (2)	O10B—O10A—Mo3^i^	68.9 (5)
O2—Mo1—O2^ii^	85.9 (3)	As1—O10A—Mo3^i^	121.8 (5)
O5—Mo1—O2^ii^	82.5 (2)	Mo2—O10A—Mo3^i^	99.2 (4)
O3—Mo1—O1	164.9 (3)	O10A^i^—O10B—O10A	118.4 (9)
O4—Mo1—O1	87.7 (2)	O10A^i^—O10B—As1	60.9 (5)
O2—Mo1—O1	73.8 (2)	O10A—O10B—As1	60.5 (5)
O5—Mo1—O1	83.8 (2)	O10A^i^—O10B—O12B^i^	121.6 (8)
O2^ii^—Mo1—O1	71.36 (19)	O10A—O10B—O12B^i^	119.9 (8)
O7—Mo2—O12B	113.9 (5)	As1—O10B—O12B^i^	159.0 (8)
O7—Mo2—O9	100.7 (3)	O10A^i^—O10B—Mo2^i^	70.8 (5)
O12B—Mo2—O9	88.5 (5)	O10A—O10B—Mo2^i^	169.3 (7)
O7—Mo2—O6	99.7 (4)	As1—O10B—Mo2^i^	125.6 (5)
O12B—Mo2—O6	144.4 (5)	O12B^i^—O10B—Mo2^i^	50.8 (5)
O9—Mo2—O6	97.0 (4)	O10A^i^—O10B—Mo3^i^	165.0 (7)
O7—Mo2—O12A	91.1 (5)	O10A—O10B—Mo3^i^	71.2 (5)
O12B—Mo2—O12A	23.4 (4)	As1—O10B—Mo3^i^	122.5 (5)
O9—Mo2—O12A	98.1 (5)	O12B^i^—O10B—Mo3^i^	49.3 (5)
O6—Mo2—O12A	159.5 (5)	Mo2^i^—O10B—Mo3^i^	98.7 (3)
O7—Mo2—O4	92.9 (3)	Cu1—O11—H1W	117 (2)
O12B—Mo2—O4	83.0 (4)	O12B—O12A—Mo3	64.0 (14)
O9—Mo2—O4	166.0 (2)	O12B—O12A—Mo2	63.0 (14)
O6—Mo2—O4	83.8 (3)	Mo3—O12A—Mo2	123.3 (7)
O12A—Mo2—O4	78.2 (4)	O12A—O12B—O10B^ii^	155.9 (19)
O7—Mo2—O10B^ii^	158.9 (3)	O12A—O12B—Mo3	91.9 (16)
O12B—Mo2—O10B^ii^	46.8 (5)	O10B^ii^—O12B—Mo3	84.1 (7)
O9—Mo2—O10B^ii^	88.6 (3)	O12A—O12B—Mo2	93.7 (16)
O6—Mo2—O10B^ii^	98.0 (4)	O10B^ii^—O12B—Mo2	82.4 (7)
O12A—Mo2—O10B^ii^	68.6 (5)	Mo3—O12B—Mo2	158.4 (8)
O4—Mo2—O10B^ii^	77.5 (3)		

Symmetry codes: (i) −*y*, *x*−*y*, *z*; (ii) −*x*+*y*, −*x*, *z*; (iii) *y*, *x*, −*z*+1/2; (iv) *y*, −*x*+*y*, −*z*; (v) *x*−*y*, *x*, −*z*.

### Hydrogen-bond geometry (Å, °)

**Table:**

*D*—H···*A*	*D*—H	H···*A*	*D*···*A*	*D*—H···*A*
N2—H2A···O6^vi^	0.86	2.27	3.097 (13)	162
O11—H1W···O3^iii^	0.84 (8)	2.69 (11)	2.860 (10)	94 (8)

Symmetry codes: (vi) *x*+1/3, *x*−*y*+2/3, *z*+1/6; (iii) *y*, *x*, −*z*+1/2.
